# Antegrade transpapillary biliary stent placement via endosonography‐guided choledochoduodenostomy for treatment of recurrent cholangitis

**DOI:** 10.1111/den.14252

**Published:** 2022-02-20

**Authors:** Roy L. J. van Wanrooij, Rogier P. Voermans, Paul Fockens

**Affiliations:** ^1^ Department of Gastroenterology and Hepatology Amsterdam Gastroenterology Endocrinology Metabolism Amsterdam UMC Free University Amsterdam The Netherlands; ^2^ Department of Gastroenterology and Hepatology Amsterdam Gastroenterology Endocrinology Metabolism Amsterdam UMC University of Amsterdam Amsterdam The Netherlands

## Abstract

Watch a video of this article.

## Brief Explanation

**Figure 1:**
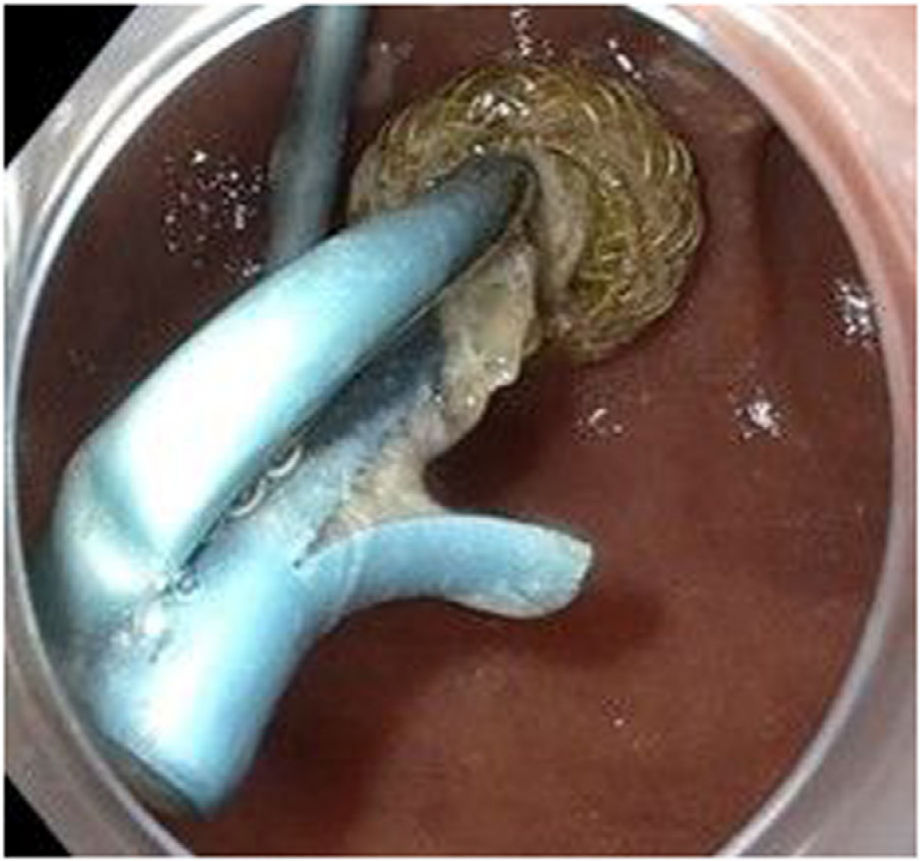
Occluded lumen‐apposing metal stent with plastic stents after endosonography‐guided choledochoduodenostomy.

An 87‐year‐old woman presented with jaundice and gastric outlet obstruction caused by an ampullary carcinoma. An endosonography‐guided choledochoduodenostomy (EUS‐CDS) and gastroenterostomy (EUS‐GE) using lumen‐apposing metal stents (LAMS) were performed. Unfortunately, the patient experienced three bouts of cholangitis at 2, 6, and 9 weeks. Food remnants were each time endoscopically removed from the LAMS and bile ducts. Despite placement of plastic pigtail stents (PPS) through the LAMS to prevent blockage of the LAMS, cholangitis recurred (Fig. 1). PPS and the LAMS were removed after the second recurrence of cholangitis and a transpapillary biliary self‐expandable metal stent (SEMS) was placed via the EUS‐CDS (Video S1). No stents were replaced through the fistulous tract to let it close naturally. Adequate drainage of the distal bile duct was now established, and the port of entrance of food remnants into the bile duct was closed off. Since the procedure 5 months ago, serum bilirubin levels have normalized, and the patient has been clinically well.

**Figure 2:**
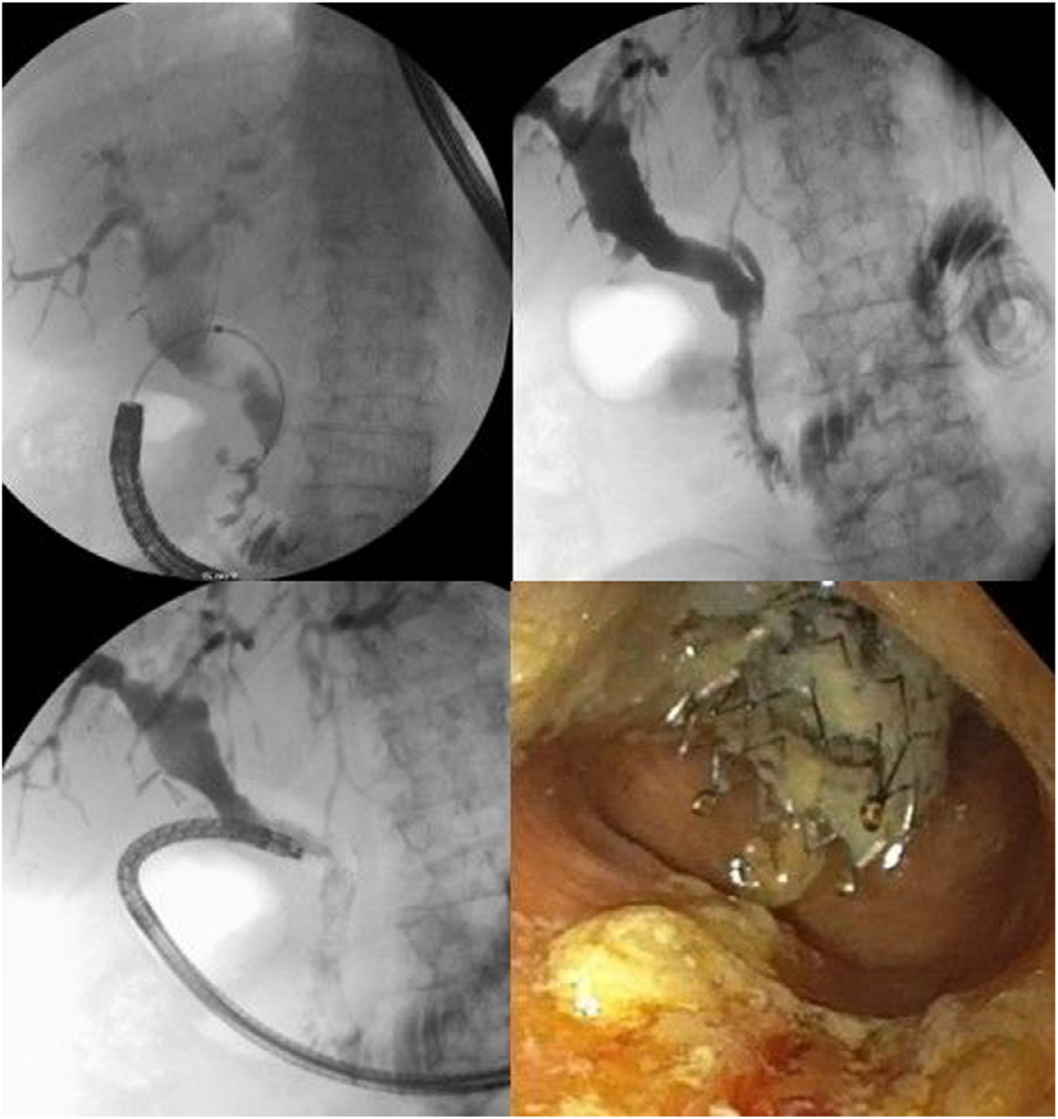
Fluoroscopic image (top left) depicts canulation of the common bile duct via the choledochoduodenostomy fistulous tract with a catheter, and traversing of the distal biliary stricture with a guidewire. Fluoroscopic image (top right) illustrating adequate flow of contrast injected via the choledochoduodenostomy fistulous tract through the metal biliary stent into the duodenum towards the gastroenterostomy. Fluoroscopic image (bottom left) depicting the nasogastric scoop inside the bile duct. Endoscopic image (bottom right) of the common bile duct and the metal biliary stent during cholangioscopy.

Endosonography‐CDS is utilized when ERCP fails and has a high technical and clinical success. Stent dysfunction is, however, not uncommon and reinterventions are reported in 11% of cases when using LAMS.^1^ Stent dysfunction may occur due to food impaction, or alternatively, due to sump syndrome, a well‐known complication of surgical choledochoduodenostomy, where the distal bile duct is poorly drained and serves as a reservoir for static bile and debris, leading to cholangitis.^2^ Placement of PPS through the LAMS has been successfully utilized as rescue therapy in these cases, but failed in our patient.^3^ Antegrade placement of a transpapillary SEMS via the EUS‐CDS, with subsequent removal of the LAMS, may serve as a new strategy in this scenario (Fig. 2). Future studies should address the optimal EUS‐guided biliary drainage strategy, especially in patients with gastric outlet obstruction, both treated and untreated.

## Conflict of Interest

Author R.P.V. has received research grant and consulting fees from Boston Scientific. P.F. has received consulting fees from Olympus/Cook Medical. The other author declares no conflict of interest for this article.

## Supporting information


**Video S1** Endoscopic antegrade biliary stent placement via endosonography‐guided choledochoduodenostomy for treatment of recurrent cholangitis.
